# Studies in Hawaiian Diptera I: New Distributional Records for Endemic *Asteia* (Asteiidae)

**DOI:** 10.3897/BDJ.2.e1010

**Published:** 2014-02-10

**Authors:** Patrick M O'Grady, Karl Nicholas Magnacca

**Affiliations:** † UC Berkeley, Berkeley, United States of America; ‡ Oahu Army Natural Resources Program, Schofield Barracks, Hawaii, United States of America; § Research Affiliate, B. P. Bishop Museum, Honolulu, United States of America

**Keywords:** Hawaiian Islands, Diptera, Asteiidae, Asteia, distributions, new island records

## Abstract

New island records are reported for five species of *Asteia* endemic to the Hawaiian Islands (*Asteia hawaiiensis*, *Asteia mauiensis*, *Asteia molokaiensis*, *Asteia palikuensis*, *Asteia sabroskyi*). These new records expand our understanding of distributions in *Asteia*, change the percentage of single island endemics from 78% to 33%, and have significance in how we view the process of diversification acting in this lineage. We also present a list of the known rearing records for two species in this group. *Asteia montgomeryi* has been recorded from *Erythrina* and *Asteia sabroskyi* has been reared from *Pisonia*, *Urera*, *Charpentiera* and *Hibiscadelphus*.

## Introduction

Research on the Diptera of the Hawaiian Islands dates to the last half of the 19th century and was first summarized in the Fauna Hawaiiensis (1899-1910) series (Sharp 1899). Zimmerman's multivolume Insects of Hawaii series (1948-2003) has also added greatly to our knowledge of the endemic and introduced insect species present in Hawaii (Zimmerman 1948). The Proceedings of the Hawaiian Entomological Society and the Records of the B. P. Bishop Museum are two other venues that were used by Hawaiian entomologists to publish shorter works or updates on new species, new invaders or new island records. It was from these works that the Bishop Museum created a comprehensive checklist of the Terrestrial Arthropods of the Hawaiian Islands (Nishida 2002). My lab has been conducting collections and surveys in Hawaii as part of various projects for over 15 years. As a result, we have many new species and new island records and have generated a number of new identification tools for several fly families. The goal of this multipart series is to publish these data in an accessible format to facilitate further research on Hawaiian Diptera. Future papers will focus on new island records and identification tools for *Dicranomyia*, a description of a new species of *Procancae* with a revised key to the genus, and new island records for *Scatella*.

Asteiidae are a small family of acalyptrate flies, containing approximately 130 species worldwide. There are 11 genera in the family but over half of the described species belong to a single genus, *Asteia* (Sabrosky 1956, Sabrosky 1957, Sabrosky 1987). The ecological associations of this group are unclear, although members of this family have been reared from a variety of substrates, including fungi, frass, bleeding trees and dried stems (Sabrosky 1987).

The most recent revision of the Hawaiian Asteiidae (Hardy and Delfinado 1980), lists three genera, *Asteia*, *Bryania*, and *Loewimyia*, as well as a comprehensive key to Hawaiian members of this family. *Bryania* and *Loewimyia* each contain a single species from Hawaii and are uncommon in collections. Hardy and Delfinado (1980) considered *Bryania bipunctata*, the only species described in this genus, an endemic species from the Northwest Hawaiian Islands. The relationship of *Bryania* to the other Hawaiian asteiids remains unclear. They described *Loewimyia orbiculata* as a new species from Oahu but did not comment on whether or not they considered it to be endemic (Hardy and Delfinado 1980). The genus *Loewimyia* is known only from one other species found only in North America so it may be a migrant. Members of the genus *Asteia* are the largest and most commonly collected asteiids, comprising nine of the eleven Hawaiian asteiid species (Table 1).

Many Hawaiian radiations are characterized by a high degree of single island endemism. For example, >95% of the ~300 species Hawaiian *Campsicnemus* (Diptera: Dolichopodidae) are single island endemics (Evenhuis 2008) and approximately 85% of the 1000 Hawaiian *Drosophila* (Diptera: Drosophilidae) are also found on just one island (Evenhuis 2008, O'Grady et al. 2009). In contrast, smaller groups like the genus *Dicranomyia* (Diptera: Limoniidae) have less than 30% of species endemic to a single island (Nitta and O’Grady 2008, Goodman and O'Grady 2013). According to the latest revision of the Hawaiian *Asteia* (Hardy and Delfinado 1980), seven of the nine described species (78%) are single island endemics. However, recent collections demonstrate that only three of the nine (33%) known species are endemic to a single island (Table 1).

## Materials and methods

### Literature Review

We have reviewed the literature for the Hawaiian *Asteia* and have attempted to include infomation on both the taxonomic history of the species and all occurrences of the endemic taxa in the literature, including the original descriptions, subsequent revisions, additional descriptive notes, range expansions and new island records and catalogs. The original authors of most of the species covered here designated holotypes, allotypes and paratypes in the original descriptions. We list the type, paratypes and other material examined. We have attempted to include as much collection data from the original publications as possible. In some cases label data were elaborated via examination of material deposited in the B. P. Bishop Museum or the University of Hawaii Entomological Collection.

### Collection Methods

All material was obtained from general sweeping of vegetation, seeps and streams. Samples were preserved in 95% ethanol (ETOH) and transported to UC Berkeley for identification. Key and descriptions by Hardy and Delfinado (1980) were used to identify the Hawaiian *Asteia*. Historical specimens examined in this study are deposited in the University of Hawaii Entomology Collection (UHEC) or the B. P. Bishop Museum (BPBM). All material collected by the authors was preserved in 95% ETOH and deposited in the University of California’s Essig Museum of Entomology (UCB).

## Checklists

### Checklist of Hawaiian *Asteia* (Diptera: Asteiidae)

#### Asteia aberrans

Hardy & Delfinado, 1980

Asteia aberrans Hardy & Delfinado, 1980: 233

##### Materials

- **Type status:** Holotype. **Occurrence:** recordedBy: JW Beardsley; individualCount: 1; sex: female; **Location:** islandGroup: Hawaiian Islands; island: Northwest Hawaiian Islands; verbatimLocality: Nihoa; **Event:** verbatimEventDate: 23-24.ix.1964; **Record Level:** institutionCode: BPBM
- **Type status:** Paratype. **Occurrence:** recordedBy: JW Beardsley; individualCount: 2; sex: female; **Location:** islandGroup: Hawaiian Islands; island: Northwest Hawaiian Islands; verbatimLocality: Nihoa; **Event:** verbatimEventDate: 23-24.ix.1964; **Record Level:** institutionCode: BPBM
- **Type status:** Paratype. **Occurrence:** individualCount: 1; sex: male; **Location:** islandGroup: Hawaiian Islands; island: Northwest Hawaiian Islands; verbatimLocality: Necker; **Event:** verbatimEventDate: 26-27.ix.1964; **Record Level:** institutionCode: UHEC
- **Type status:** Other material. **Occurrence:** recordedBy: WC Gagne; individualCount: 4; **Location:** islandGroup: Hawaiian Islands; island: Northwest Hawaiian Islands; verbatimLocality: Nihoa, E Palm Valley, in moist shelter cave; verbatimElevation: 150m; **Event:** eventDate: 27.iv.1983; **Record Level:** collectionID: 1983.184; institutionCode: BPBM
- **Type status:** Other material. **Occurrence:** recordedBy: J. Strazanac; individualCount: 1; **Location:** islandGroup: Hawaiian Islands; island: Northwest Hawaiian Islands; verbatimLocality: Nihoa, Miller V, yellow pan trap in low shrubs; verbatimElevation: 100 ft; **Event:** eventDate: 22.vi.1990; **Record Level:** institutionCode: BPBM

##### Ecological interactions

###### Native status

endemic

##### Distribution

HAWAIIAN ISLANDS: Northwest Hawaiian Islands (Necker, Nihoa)

##### Notes

Hardy and Delfinado (1980) [original description; illustration of male genitalia (right lateral), head (lateral), wing]; Sabrosky (1989) [Australasian and Oceanian Catalog]; Nishida (2002) [Hawaiian Terrestrial Arthropod Checklist]; Evenhuis (2011) [http://hbs.bishopmuseum.org/aocat/asteiidae.html, Australasian and Oceanian Catalog, online version].

#### Asteia apicalis

Grimshaw, 1901

Asteia apicalis Grimshaw, 1901: 73

##### Materials

- **Type status:** Holotype. **Occurrence:** recordedBy: no collector given; individualCount: 1; sex: male; **Location:** islandGroup: Hawaiian Islands; island: Hawaii; verbatimLocality: Kilauea; **Event:** verbatimEventDate: vii.1895; **Record Level:** institutionCode: BMNH
- **Type status:** Paratype. **Occurrence:** recordedBy: no collector given; individualCount: 3; sex: male; **Location:** islandGroup: Hawaiian Islands; island: Hawaii; verbatimLocality: Kilauea; **Event:** verbatimEventDate: vii.1895; **Record Level:** institutionCode: BMNH
- **Type status:** Other material. **Occurrence:** recordedBy: OH Swezey; individualCount: 1; **Location:** islandGroup: Hawaiian Islands; island: Hawaii; verbatimLocality: Kilauea; **Identification:** identifiedBy: DE Hardy; **Event:** eventDate: 27.vi.1917; **Record Level:** institutionCode: BPBM
- **Type status:** Other material. **Occurrence:** recordedBy: OH Swezey; individualCount: 1; **Location:** islandGroup: Hawaiian Islands; island: Hawaii; verbatimLocality: Upper Hamakua Ditch Trail; **Identification:** identifiedBy: DE Hardy; **Event:** eventDate: 3.ix.1919; **Record Level:** institutionCode: BPBM
- **Type status:** Other material. **Occurrence:** recordedBy: OH Swezey; individualCount: 5; **Location:** islandGroup: Hawaiian Islands; island: Hawaii; verbatimLocality: Upper Hamakua Ditch Trail; **Identification:** identifiedBy: DE Hardy; **Event:** eventDate: 1-7.x.1929; **Record Level:** institutionCode: BPBM
- **Type status:** Other material. **Occurrence:** recordedBy: WW Wirth, CJ Davis; sex: female; **Location:** islandGroup: Hawaiian Islands; island: Hawaii; verbatimLocality: Kilauea, Hawaii National Park; **Event:** verbatimEventDate: viii.1946; **Record Level:** institutionCode: USNM
- **Type status:** Other material. **Occurrence:** recordedBy: WW Wirth, CJ Davis; sex: female; **Location:** islandGroup: Hawaiian Islands; island: Hawaii; verbatimLocality: Kilauea, Hawaii National Park; **Event:** verbatimEventDate: ix.1946; **Record Level:** institutionCode: USNM
- **Type status:** Other material. **Occurrence:** recordedBy: JW Beardsley; individualCount: 1; **Location:** islandGroup: Hawaiian Islands; island: Hawaii; verbatimLocality: Kilauea, light trap; **Event:** eventDate: viii.1958; **Record Level:** institutionCode: BPBM
- **Type status:** Other material. **Occurrence:** recordedBy: FG Howarth; individualCount: 28; **Location:** islandGroup: Hawaiian Islands; island: Hawaii; verbatimLocality: Kilauea For. Res. IBP Study Site; verbatimElevation: 1586 m; **Identification:** identifiedBy: DE Hardy; **Event:** eventDate: 2.vii.1971; **Record Level:** institutionCode: BPBM
- **Type status:** Other material. **Occurrence:** recordedBy: KN Magnacca; individualCount: 2; **Location:** islandGroup: Hawaiian Islands; island: Maui; verbatimLocality: Kaupo Trail, Haleakala National Park, on and under Acacia koa; verbatimElevation: 5500 ft; **Identification:** identifiedBy: PM O'Grady; **Event:** verbatimEventDate: 2.viii.2007; **Record Level:** institutionID: EMEC7356000; institutionCode: EMEC; collectionCode: 07-0735
- **Type status:** Other material. **Occurrence:** recordedBy: no collector given; individualCount: 1; **Location:** islandGroup: Hawaiian Islands; island: Hawaii; verbatimLocality: Kau; verbatimElevation: 4000 ft; **Identification:** identifiedBy: DE Hardy; **Event:** eventDate: no date given; **Record Level:** institutionCode: BPBM

##### Ecological interactions

###### Native status

endemic

##### Distribution

HAWAIIAN ISLANDS: Maui, Hawaii

##### Notes

Grimshaw (1901) [original description]; Sabrosky (1947) [redescription]; Hardy and Delfinado (1980) [redescription; illustration of male genitalia (terminal), male genitalia (right lateral), antenna (lateral)]; Sabrosky (1989) [Australasian and Oceanian Catalog]; Nishida (2002) [Hawaiian Terrestrial Arthropod Checklist]; Evenhuis (2011) [http://hbs.bishopmuseum.org/aocat/asteiidae.html, Australasian and Oceanian Catalog, online version].

#### Asteia hawaiiensis

Grimshaw, 1901

Asteia hawaiiensis Grimshaw, 1901: 73

##### Materials

- **Type status:** Holotype. **Occurrence:** recordedBy: no collector given; individualCount: 1; sex: male; **Location:** islandGroup: Hawaiian Islands; island: Hawaii; verbatimLocality: Kona; verbatimElevation: 3500-400 ft; **Event:** verbatimEventDate: vi-vii.1892; **Record Level:** institutionCode: BMNH
- **Type status:** Paratype. **Occurrence:** recordedBy: no collector given; individualCount: 4; **Location:** islandGroup: Hawaiian Islands; island: Hawaii; verbatimLocality: Kona; verbatimElevation: 3500-400 ft; **Event:** verbatimEventDate: vi-vii.1892; **Record Level:** institutionCode: BMNH
- **Type status:** Paratype. **Occurrence:** recordedBy: no collector given; individualCount: 1; sex: female; **Location:** islandGroup: Hawaiian Islands; island: Hawaii; verbatimLocality: Olaa; **Event:** verbatimEventDate: xi.1896; **Record Level:** institutionCode: BMNH
- **Type status:** Other material. **Occurrence:** recordedBy: OH Swezey; individualCount: 1; **Location:** islandGroup: Hawaiian Islands; island: Hawaii; verbatimLocality: Glenwood; **Identification:** identifiedBy: EH Bryan; **Event:** eventDate: 3.ii.1919; **Record Level:** institutionCode: BPBM
- **Type status:** Other material. **Occurrence:** recordedBy: OH Swezey; individualCount: 11; **Location:** islandGroup: Hawaiian Islands; island: Hawaii; verbatimLocality: S. Kona; **Identification:** identifiedBy: EH Bryan; **Event:** eventDate: 8-20.viii.1919; **Record Level:** institutionCode: BPBM
- **Type status:** Other material. **Occurrence:** recordedBy: WC Gagne, W. Mull; individualCount: 6; **Location:** islandGroup: Hawaiian Islands; island: Hawaii; verbatimLocality: Olaa Forest; verbatimElevation: 3200 ft; **Identification:** identifiedBy: K Arakaki; **Event:** eventDate: 22.vi.1985; **Record Level:** institutionCode: BPBM
- **Type status:** Other material. **Occurrence:** recordedBy: KN Magnacca; individualCount: 1; **Location:** islandGroup: Hawaiian Islands; island: Maui; verbatimLocality: Puu Kukui, Kahanaiki Gulch, sweeping veg and ground; verbatimElevation: 2100 ft; **Identification:** identifiedBy: PM O'Grady; **Event:** verbatimEventDate: 7.viii.2007; **Record Level:** institutionID: EMEC7356001; institutionCode: EMEC; collectionCode: 07-0813
- **Type status:** Other material. **Occurrence:** recordedBy: no collector given; individualCount: 1; **Location:** islandGroup: Hawaiian Islands; island: Hawaii; verbatimLocality: Kau; verbatimElevation: 4000 ft; **Identification:** identifiedBy: EH Bryan; **Event:** eventDate: no date given; **Record Level:** institutionCode: BPBM

##### Ecological interactions

###### Native status

endemic

##### Distribution

HAWAIIAN ISLANDS: Maui, Hawaii

##### Notes

Grimshaw (1901) [original description]; Hardy and Delfinado (1980) [redescription; illustration of whole fly (lateral), wing, antenna (lateral), male genitalia (left lateral)]; Sabrosky (1989) [Australasian and Oceanian Catalog]; Nishida (2002) [Hawaiian Terrestrial Arthropod Checklist]; Evenhuis (2011) [http://hbs.bishopmuseum.org/aocat/asteiidae.html, Australasian and Oceanian Catalog, online version].

#### Asteia mauiensis

Hardy & Delfinado, 1980

Asteia mauiensis Hardy & Delfinado, 1980: 238

##### Materials

- **Type status:** Holotype. **Occurrence:** recordedBy: R Namba; individualCount: 1; sex: female; **Location:** islandGroup: Hawaiian Islands; island: Maui; verbatimLocality: Waikamoi; verbatimElevation: 4000 ft; **Event:** verbatimEventDate: vii.1956; **Record Level:** institutionCode: BPBM
- **Type status:** Paratype. **Occurrence:** recordedBy: OH Swezey, HT Spieth, R Namba, DE Hardy; individualCount: 4; **Location:** islandGroup: Hawaiian Islands; island: Maui; verbatimLocality: Waikamoi; verbatimElevation: 4000 ft; **Event:** verbatimEventDate: i.1926-vii.1965; **Record Level:** institutionCode: UHEC
- **Type status:** Paratype. **Occurrence:** recordedBy: DE Hardy; individualCount: 1; sex: female; **Location:** islandGroup: Hawaiian Islands; island: Maui; verbatimLocality: Iao Valley; verbatimElevation: 1500 ft; **Event:** verbatimEventDate: vi.1952; **Record Level:** institutionCode: UHEC
- **Type status:** Paratype. **Occurrence:** recordedBy: CR Joyce; individualCount: 3; **Location:** islandGroup: Hawaiian Islands; island: Maui; verbatimLocality: Puu Kukui; **Event:** verbatimEventDate: vii.1963; **Record Level:** institutionCode: USNM
- **Type status:** Paratype. **Occurrence:** recordedBy: DE Hardy; individualCount: 1; sex: male; **Location:** islandGroup: Hawaiian Islands; island: Maui; verbatimLocality: Waikamoi; verbatimElevation: 4000 ft; **Event:** verbatimEventDate: 6.vii.1966; **Record Level:** institutionCode: BPBM
- **Type status:** Other material. **Occurrence:** recordedBy: KN Magnacca; individualCount: 2; **Location:** islandGroup: Hawaiian Islands; island: Maui; verbatimLocality: Kaupo Trail, Haleakala National Park, sweeping Lobelia hypoleuca; verbatimElevation: 6100 ft; **Identification:** identifiedBy: PM O'Grady; **Event:** verbatimEventDate: 2.viii.2007; **Record Level:** institutionID: EMEC7356002; institutionCode: EMEC; collectionCode: 07-0726
- **Type status:** Other material. **Occurrence:** recordedBy: PM O'Grady, KN Magnacca, RT Lapoint, GM Bennett; individualCount: 1; **Location:** islandGroup: Hawaiian Islands; island: Maui; verbatimLocality: Heed Trail, Waikamoi Forest Preserve; **Identification:** identifiedBy: PM O'Grady; **Event:** verbatimEventDate: 31.vii.2007; **Record Level:** institutionID: EMEC7356003; institutionCode: EMEC; collectionCode: 389
- **Type status:** Other material. **Occurrence:** recordedBy: GM Bennett; individualCount: 1; **Location:** islandGroup: Hawaiian Islands; island: Oahu; verbatimLocality: Makapuu Stream; **Identification:** identifiedBy: PM O'Grady; **Event:** verbatimEventDate: viii.2009; **Record Level:** institutionID: EMEC7356004; institutionCode: EMEC; collectionCode: 545.L

##### Ecological interactions

###### Native status

endemic

##### Distribution

HAWAIIAN ISLANDS: Oahu, Maui

##### Notes

Hardy and Delfinado (1980) [original description; illustration of female abdomen (lateral), female abdomen (lateral), male genitalia (left lateral)]; Sabrosky (1989) [Australasian and Oceanian Catalog]; Nishida (2002) [Hawaiian Terrestrial Arthropod Checklist]; Evenhuis (2011) [http://hbs.bishopmuseum.org/aocat/asteiidae.html, Australasian and Oceanian Catalog, online version].

#### Asteia molokaiensis

Hardy & Delfinado, 1980

Asteia molokaiensis Hardy & Delfinado, 1980: 238

##### Materials

- **Type status:** Holotype. **Occurrence:** recordedBy: D Gubler; individualCount: 1; sex: female; **Location:** islandGroup: Hawaiian Islands; island: Molokai; verbatimLocality: Puu Kolekole; verbatimElevation: 3600 ft; **Event:** verbatimEventDate: 20.vii.1964; **Record Level:** institutionCode: BPBM
- **Type status:** Paratype. **Occurrence:** recordedBy: DE Hardy, M Tamashiro; individualCount: 4; sex: female; **Location:** islandGroup: Hawaiian Islands; island: Molokai; verbatimLocality: Puu Kolekole; verbatimElevation: 3600 ft; **Event:** verbatimEventDate: vi.1952-viii.1964; **Record Level:** institutionCode: UHEC
- **Type status:** Other material. **Occurrence:** recordedBy: BH Gagne; individualCount: 1; sex: female; **Location:** islandGroup: Hawaiian Islands; island: Lanai; verbatimLocality: Lanaihale; verbatimElevation: 914 m; **Identification:** identifiedBy: K Arakaki; **Event:** eventDate: 20.v.1980; **Record Level:** institutionCode: BPBM
- **Type status:** Other material. **Occurrence:** recordedBy: KN Magnacca; individualCount: 1; **Location:** islandGroup: Hawaiian Islands; island: Maui; verbatimLocality: Kaupo Trail, Haleakala National Park, sweeping veg and ground; verbatimElevation: 5500 ft; **Identification:** identifiedBy: PM O'Grady; **Event:** verbatimEventDate: 2.viii.2007; **Record Level:** institutionID: EMEC7356005; institutionCode: EMEC; collectionCode: 07-0755
- **Type status:** Other material. **Occurrence:** recordedBy: KN Magnacca; individualCount: 1; **Location:** islandGroup: Hawaiian Islands; island: Maui; verbatimLocality: Makawao Forest Reserve, on and under Clermontia; verbatimElevation: 4500 ft; **Identification:** identifiedBy: PM O'Grady; **Event:** verbatimEventDate: 6.viii.2007; **Record Level:** institutionID: EMEC7356006; institutionCode: EMEC; collectionCode: 07-0808

##### Ecological interactions

###### Native status

endemic

##### Distribution

HAWAIIAN ISLANDS: Molokai, Maui

##### Notes

Hardy and Delfinado (1980) [original description; illustration of female abdomen (lateral)]; Sabrosky (1989) [Australasian and Oceanian Catalog]; Nishida (2002) [Hawaiian Terrestrial Arthropod Checklist]; Evenhuis (2011) [http://hbs.bishopmuseum.org/aocat/asteiidae.html, Australasian and Oceanian Catalog, online version].

#### Asteia montgomeryi

Hardy, 1980

Asteia montgomeryi Hardy, 1980: 239

##### Materials

- **Type status:** Holotype. **Occurrence:** recordedBy: WB Heed; individualCount: 1; sex: male; **Location:** islandGroup: Hawaiian Islands; island: Hawaii; verbatimLocality: Puuwaawaa; verbatimElevation: 2000 ft; **Event:** verbatimEventDate: 16.ii.1973; **Record Level:** institutionCode: BPBM
- **Type status:** Paratype. **Occurrence:** recordedBy: WB Heed; individualCount: 1; sex: male; **Location:** islandGroup: Hawaiian Islands; island: Hawaii; verbatimLocality: Puuwaawaa; verbatimElevation: 2000 ft; **Event:** verbatimEventDate: 16.ii.1973; **Record Level:** institutionCode: UHEC

##### Ecological interactions

###### Feeds on

This species has been reared from rotting stems of *Erythrina sandwichensis* Degener (Hawaiian Islands: Hawaii, Puuwaawaa, 2000 ft., WB Heed and SL Montgmery, iv-vi.1973)

###### Native status

endemic

##### Distribution

HAWAIIAN ISLANDS: Hawaii

##### Notes

Hardy and Delfinado (1980) [original description; illustration of male genitalia (terminal), antenna (lateral)]; Sabrosky (1989) [Australasian and Oceanian Catalog]; Nishida (2002) [Hawaiian Terrestrial Arthropod Checklist]; Evenhuis (2011) [http://hbs.bishopmuseum.org/aocat/asteiidae.html, Australasian and Oceanian Catalog, online version].

#### Asteia nudiseta

Sabrosky, 1947

Asteia nudiseta Sabrosky, 1947: 55

##### Materials

- **Type status:** Holotype. **Occurrence:** recordedBy: WW Wirth; individualCount: 1; sex: female; **Location:** islandGroup: Hawaiian Islands; island: Oahu; verbatimLocality: Mt. Kaala, bog at summit; verbatimElevation: 4000 ft; **Event:** verbatimEventDate: iv.1892; **Record Level:** institutionID: 58218; institutionCode: USNM
- **Type status:** Other material. **Occurrence:** recordedBy: OH Swezey; individualCount: 1; **Location:** islandGroup: Hawaiian Islands; island: Oahu; verbatimLocality: Haleauau, Suttonia; **Identification:** identifiedBy: DE Hardy; **Event:** eventDate: 13.iii.1932; **Record Level:** institutionCode: BPBM
- **Type status:** Other material. **Occurrence:** recordedBy: JW Beardsley; individualCount: 6; **Location:** islandGroup: Hawaiian Islands; island: Oahu; verbatimLocality: Puu Kanehoa, at light; **Identification:** identifiedBy: DE Hardy; **Event:** eventDate: 10.viii.1959; **Record Level:** institutionCode: BPBM
- **Type status:** Other material. **Occurrence:** recordedBy: A Asquith; individualCount: 5; **Location:** islandGroup: Hawaiian Islands; island: Oahu; verbatimLocality: Mt. Kaala summit, ex Melicope; **Identification:** identifiedBy: K Arakaki; **Event:** eventDate: 13.vi.1994; **Record Level:** institutionCode: BPBM
- **Type status:** Paratype. **Occurrence:** recordedBy: JC Bridwell; individualCount: 1; sex: female; **Location:** islandGroup: Hawaiian Islands; island: Oahu; verbatimLocality: Oahu; **Event:** verbatimEventDate: no date given; **Record Level:** institutionCode: USNM

##### Ecological interactions

###### Native status

endemic

##### Distribution

HAWAIIAN ISLANDS: Oahu

##### Notes

Sabrosky (1947) [original description]; Hardy and Delfinado (1980) [redescription; illustration of female abdomen (lateral), antenna (lateral), male genitalia (left lateral)]; Sabrosky (1989) [Australasian and Oceanian Catalog]; Nishida (2002) [Hawaiian Terrestrial Arthropod Checklist]; http://hbs.bishopmuseum.org/aocat/asteiidae.html; Evenhuis and Eldredge (2004); Evenhuis (2011) [http://hbs.bishopmuseum.org/aocat/asteiidae.html, Australasian and Oceanian Catalog, online version].

#### Asteia palikuensis

Hardy & Delfinado, 1980

Asteia palikuensis Hardy & Delfinado, 1980: 242

##### Materials

- **Type status:** Holotype. **Occurrence:** recordedBy: LH Throckmorton; individualCount: 1; sex: male; **Location:** islandGroup: Hawaiian Islands; island: Maui; verbatimLocality: Paliku, Haleakala Crater; verbatimElevation: 6500 ft; **Event:** verbatimEventDate: 28.vii.1964; **Record Level:** institutionCode: BPBM
- **Type status:** Paratype. **Occurrence:** recordedBy: DE Hardy, CR Joyce, D Gubler, JW Beardsley, LH Throckmorton, M Tamashiro; individualCount: 158; **Location:** islandGroup: Hawaiian Islands; island: Maui; verbatimLocality: Paliku, Haleakala Crater; verbatimElevation: 6500 ft; **Event:** verbatimEventDate: vi.1952-viii.1965; **Record Level:** institutionCode: UHEC
- **Type status:** Paratype. **Occurrence:** recordedBy: DE Hardy; **Location:** islandGroup: Hawaiian Islands; island: Maui; verbatimLocality: Holua; verbatimElevation: 6500 ft; **Event:** verbatimEventDate: vi.1953; **Record Level:** institutionCode: USNM, BMNH
- **Type status:** Paratype. **Occurrence:** recordedBy: LH Throckmorton; individualCount: 1; sex: female; **Location:** islandGroup: Hawaiian Islands; island: Maui; verbatimLocality: Paliku, Haleakala Crater; verbatimElevation: 6500 ft; **Event:** verbatimEventDate: 28.vii.1964; **Record Level:** institutionCode: BPBM
- **Type status:** Paratype. **Occurrence:** recordedBy: JW Beardsley; **Location:** islandGroup: Hawaiian Islands; island: Maui; verbatimLocality: Kaupo Trail; verbatimElevation: 5500 - 6500 ft; **Event:** verbatimEventDate: 22.vii.1965; **Record Level:** institutionCode: USNM, BMNH
- **Type status:** Paratype. **Occurrence:** recordedBy: JW Beardsley; **Location:** islandGroup: Hawaiian Islands; island: Maui; verbatimLocality: Oili Puu; **Event:** verbatimEventDate: 23.vii.1965; **Record Level:** institutionCode: USNM, BMNH
- **Type status:** Other material. **Occurrence:** recordedBy: WA Steffan; individualCount: 1; **Location:** islandGroup: Hawaiian Islands; island: Hawaii; verbatimLocality: Kilauea For. Res. IBP Malaise Trap #2; verbatimElevation: 1586 m; **Identification:** identifiedBy: K Arakaki; **Event:** eventDate: 16-23.viii.1971; **Record Level:** institutionCode: BPBM
- **Type status:** Other material. **Occurrence:** recordedBy: T Parman; individualCount: 1; **Location:** islandGroup: Hawaiian Islands; island: Maui; verbatimLocality: Hana Forest Reserve, stream below base camp, Cheirodendron trigynum; verbatimElevation: 2118 m; **Identification:** identifiedBy: K Arakaki; **Event:** eventDate: 10.viii.1974; **Record Level:** institutionCode: BPBM
- **Type status:** Other material. **Occurrence:** recordedBy: R Burkhart; individualCount: 8; **Location:** islandGroup: Hawaiian Islands; island: Maui; verbatimLocality: Haleakala, Paliku, malaise; verbatimElevation: 6500 ft.; **Identification:** identifiedBy: K Arakaki; **Event:** eventDate: 27.vi.1975; **Record Level:** institutionCode: BPBM
- **Type status:** Other material. **Occurrence:** recordedBy: R Burkhart; individualCount: 3; **Location:** islandGroup: Hawaiian Islands; island: Maui; verbatimLocality: Haleakala NP, Kaupo Gap, malaise; verbatimElevation: 6100 ft.; **Identification:** identifiedBy: K Arakaki; **Event:** eventDate: 3.vii.1975; **Record Level:** institutionCode: BPBM
- **Type status:** Other material. **Occurrence:** recordedBy: R Burkhart; individualCount: 1; **Location:** islandGroup: Hawaiian Islands; island: Maui; verbatimLocality: Haleakala, Paliku Cabin, malaise; verbatimElevation: 6400 ft.; **Identification:** identifiedBy: K Arakaki; **Event:** eventDate: 4.vii.1975; **Record Level:** institutionCode: BPBM
- **Type status:** Other material. **Occurrence:** recordedBy: KN Magnacca; individualCount: 1; **Location:** islandGroup: Hawaiian Islands; island: Maui; verbatimLocality: Paliku, crater wall, sweeping veg and ground; verbatimElevation: 6600 ft.; **Identification:** identifiedBy: PM O'Grady; **Event:** verbatimEventDate: 1.viii.2007; **Record Level:** institutionID: EMEC7356007; institutionCode: EMEC; collectionCode: 07-0716
- **Type status:** Other material. **Occurrence:** recordedBy: KN Magnacca; individualCount: 1; **Location:** islandGroup: Hawaiian Islands; island: Lanai; verbatimLocality: Munro Trail, sweeping veg and ground; verbatimElevation: 2800 ft; **Identification:** identifiedBy: PM O'Grady; **Event:** verbatimEventDate: 30.vii.2007; **Record Level:** institutionID: EMEC7356008; institutionCode: EMEC; collectionCode: 07-0691
- **Type status:** Other material. **Occurrence:** recordedBy: KN Magnacca; individualCount: 2; **Location:** islandGroup: Hawaiian Islands; island: Maui; verbatimLocality: Kaupo Trail, Haleakala National Park, sweeping Lobelia hypoleuca; verbatimElevation: 6100 ft; **Identification:** identifiedBy: PM O'Grady; **Event:** verbatimEventDate: 2.viii.2007; **Record Level:** institutionID: EMEC7356009; institutionCode: EMEC; collectionCode: 07-0727
- **Type status:** Other material. **Occurrence:** recordedBy: KN Magnacca; individualCount: 3; **Location:** islandGroup: Hawaiian Islands; island: Maui; verbatimLocality: Paliku Cabin, on and under Cheirodendron; verbatimElevation: 6400 ft; **Identification:** identifiedBy: PM O'Grady; **Event:** verbatimEventDate: 2.viii.2007; **Record Level:** institutionID: EMEC7356010; institutionCode: EMEC; collectionCode: 07-0748
- **Type status:** Other material. **Occurrence:** recordedBy: KN Magnacca; individualCount: 6; **Location:** islandGroup: Hawaiian Islands; island: Maui; verbatimLocality: Kaupo Trail, Haleakala National Park, sweeping veg and ground; verbatimElevation: 5500 ft; **Identification:** identifiedBy: PM O'Grady; **Event:** verbatimEventDate: 4.viii.2007; **Record Level:** institutionID: EMEC7356011; institutionCode: EMEC; collectionCode: 07-0754

##### Ecological interactions

###### Native status

endemic

##### Distribution

HAWAIIAN ISLANDS: Lanai, Maui

##### Notes

Hardy and Delfinado (1980) [original description; illustration of head (anterior), head, (lateral), wing, female abdomen (lateral), male genitalia (left lateral), antenna (lateral)]; Sabrosky (1989) [Australasian and Oceanian Catalog]; Nishida (2002) [Hawaiian Terrestrial Arthropod Checklist]; Evenhuis (2011) [http://hbs.bishopmuseum.org/aocat/asteiidae.html, Australasian and Oceanian Catalog, online version].

#### Asteia sabroskyi

Hardy & Delfinado, 1980

Asteia sabroskyi Hardy & Delfinado, 1980: 244

##### Materials

- **Type status:** Holotype. **Occurrence:** recordedBy: SL Montgomery; individualCount: 1; sex: male; **Location:** islandGroup: Hawaiian Islands; island: Oahu; verbatimLocality: Mt. Tantalus; **Event:** verbatimEventDate: 1.i.1970; **Record Level:** institutionCode: BPBM
- **Type status:** Paratype. **Occurrence:** recordedBy: OH Swezey; **Location:** islandGroup: Hawaiian Islands; island: Oahu; verbatimLocality: Waiawa; **Event:** verbatimEventDate: 8.vi.1921; **Record Level:** institutionCode: USNM, BMNH
- **Type status:** Other material. **Occurrence:** recordedBy: NLH Krauss; **Location:** islandGroup: Hawaiian Islands; island: Hawaii; verbatimLocality: Waimea; **Event:** verbatimEventDate: 26.iv.1944; **Record Level:** institutionCode: UHEC
- **Type status:** Paratype. **Occurrence:** recordedBy: no collector given; **Location:** islandGroup: Hawaiian Islands; island: Oahu; verbatimLocality: Manoa; **Event:** verbatimEventDate: 12.iii.1945; **Record Level:** institutionCode: USNM, BMNH
- **Type status:** Other material. **Occurrence:** recordedBy: DE Hardy; **Location:** islandGroup: Hawaiian Islands; island: Molokai; verbatimLocality: Maunawainui Valley; **Event:** verbatimEventDate: vii.1952; **Record Level:** institutionCode: UHEC
- **Type status:** Other material. **Occurrence:** recordedBy: DE Hardy; **Location:** islandGroup: Hawaiian Islands; island: Kauai; verbatimLocality: Kokee; verbatimElevation: 3600 ft; **Event:** verbatimEventDate: vii.1953; **Record Level:** institutionCode: UHEC
- **Type status:** Other material. **Occurrence:** recordedBy: DE Hardy; **Location:** islandGroup: Hawaiian Islands; island: Kauai; verbatimLocality: Halemanu Valley; **Event:** verbatimEventDate: viii.1953; **Record Level:** institutionCode: UHEC
- **Type status:** Other material. **Occurrence:** recordedBy: DE Hardy; **Location:** islandGroup: Hawaiian Islands; island: Kauai; verbatimLocality: Kawaikoi Stream; verbatimElevation: 3700 ft; **Event:** verbatimEventDate: viii.1953; **Record Level:** institutionCode: UHEC
- **Type status:** Paratype. **Occurrence:** recordedBy: JW Beardsley; **Location:** islandGroup: Hawaiian Islands; island: Oahu; verbatimLocality: Mt. Tantalus; **Event:** verbatimEventDate: 5.v.1956; **Record Level:** institutionCode: UHEC
- **Type status:** Other material. **Occurrence:** recordedBy: DE Hardy; **Location:** islandGroup: Hawaiian Islands; island: Hawaii; verbatimLocality: Pauahi; verbatimElevation: 4300 ft; **Event:** verbatimEventDate: viii.1956; **Record Level:** institutionCode: UHEC
- **Type status:** Other material. **Occurrence:** recordedBy: WC Mitchell; **Location:** islandGroup: Hawaiian Islands; island: Hawaii; verbatimLocality: Mauna Loa Truck Trail; verbatimElevation: 4250 ft; **Event:** verbatimEventDate: xi.1956; **Record Level:** institutionCode: UHEC
- **Type status:** Paratype. **Occurrence:** recordedBy: CR Joyce; **Location:** islandGroup: Hawaiian Islands; island: Oahu; verbatimLocality: Honolulu; **Event:** verbatimEventDate: 13.i.1958; **Record Level:** institutionCode: USNM, BMNH
- **Type status:** Paratype. **Occurrence:** recordedBy: CR Joyce; **Location:** islandGroup: Hawaiian Islands; island: Oahu; verbatimLocality: Honolulu; **Event:** verbatimEventDate: 8.viii.1958; **Record Level:** institutionCode: USNM, BMNH
- **Type status:** Paratype. **Occurrence:** recordedBy: CR Joyce; **Location:** islandGroup: Hawaiian Islands; island: Oahu; verbatimLocality: Nuuanu; **Event:** verbatimEventDate: 26.i.1959; **Record Level:** institutionCode: USNM, BMNH
- **Type status:** Paratype. **Occurrence:** recordedBy: CR Joyce; **Location:** islandGroup: Hawaiian Islands; island: Oahu; verbatimLocality: Nuuanu; **Event:** verbatimEventDate: 6.iv.1959; **Record Level:** institutionCode: USNM, BMNH
- **Type status:** Paratype. **Occurrence:** recordedBy: JW Beardsley; **Location:** islandGroup: Hawaiian Islands; island: Oahu; verbatimLocality: Puu Kanehoa; **Event:** verbatimEventDate: 25.vii-10.viii.1959; **Record Level:** institutionCode: USNM, BMNH
- **Type status:** Other material. **Occurrence:** recordedBy: JW Beardsley; individualCount: 1; **Location:** islandGroup: Hawaiian Islands; island: Kauai; verbatimLocality: Kokee, light trap; **Event:** eventDate: 29.viii.1959; **Record Level:** institutionCode: BPBM
- **Type status:** Other material. **Occurrence:** recordedBy: TC Maa; individualCount: 2; **Location:** islandGroup: Hawaiian Islands; island: Oahu; verbatimLocality: Puu Palikea, Metrosideros; verbatimElevation: 2500 ft; **Identification:** identifiedBy: K Arakaki; **Event:** eventDate: 15.x.1960; **Record Level:** institutionCode: BPBM
- **Type status:** Paratype. **Occurrence:** recordedBy: CR Joyce; **Location:** islandGroup: Hawaiian Islands; island: Oahu; verbatimLocality: Nuuanu; **Event:** verbatimEventDate: 10.vii.1961; **Record Level:** institutionCode: UHEC
- **Type status:** Paratype. **Occurrence:** recordedBy: LH Throckmorton; **Location:** islandGroup: Hawaiian Islands; island: Oahu; verbatimLocality: Mt. Kaala; **Event:** verbatimEventDate: 30.vi.1963; **Record Level:** institutionCode: USNM
- **Type status:** Paratype. **Occurrence:** recordedBy: JL Gressit; **Location:** islandGroup: Hawaiian Islands; island: Oahu; verbatimLocality: Waimano Trail; verbatimElevation: 400-500m; **Event:** verbatimEventDate: 5.vii.1963; **Record Level:** institutionCode: BPBM
- **Type status:** Other material. **Occurrence:** recordedBy: DE Hardy, HT Spieth, LH Throckmorton; **Location:** islandGroup: Hawaiian Islands; island: Kauai; verbatimLocality: Mohihi Stream, Kokee; **Event:** verbatimEventDate: 27.vii.1963; **Record Level:** institutionCode: UHEC
- **Type status:** Other material. **Occurrence:** recordedBy: LH Throckmorton; **Location:** islandGroup: Hawaiian Islands; island: Hawaii; verbatimLocality: Bird Park, Kilauea; **Event:** verbatimEventDate: 24.vi.1964; **Record Level:** institutionCode: UHEC
- **Type status:** Other material. **Occurrence:** recordedBy: DE Hardy; **Location:** islandGroup: Hawaiian Islands; island: Hawaii; verbatimLocality: Hualalei; verbatimElevation: 2000 ft; **Event:** verbatimEventDate: 18.viii.1964; **Record Level:** institutionCode: UHEC
- **Type status:** Other material. **Occurrence:** recordedBy: DE Hardy, HT Spieth, LH Throckmorton; **Location:** islandGroup: Hawaiian Islands; island: Kauai; verbatimLocality: Mohihi Stream, Kokee; **Event:** verbatimEventDate: 28.viii.1964; **Record Level:** institutionCode: UHEC
- **Type status:** Other material. **Occurrence:** recordedBy: HT Spieth; **Location:** islandGroup: Hawaiian Islands; island: Hawaii; verbatimLocality: Kipuka Ki; **Event:** verbatimEventDate: 8.ix.1964; **Record Level:** institutionCode: UHEC
- **Type status:** Other material. **Occurrence:** recordedBy: JW Beardsley; **Location:** islandGroup: Hawaiian Islands; island: Maui; verbatimLocality: Ulukalapua; **Event:** verbatimEventDate: 26.v.1965; **Record Level:** institutionCode: UHEC
- **Type status:** Other material. **Occurrence:** recordedBy: CM Yoshimoto; **Location:** islandGroup: Hawaiian Islands; island: Kauai; verbatimLocality: Alakai, Kokee; verbatimElevation: 4000 ft; **Event:** verbatimEventDate: 14.ix.1965; **Record Level:** institutionCode: UHEC
- **Type status:** Paratype. **Occurrence:** recordedBy: SL Montgomery; **Location:** islandGroup: Hawaiian Islands; island: Oahu; verbatimLocality: East Makaleha; **Event:** verbatimEventDate: 7.xii.1969; **Record Level:** institutionCode: UHEC
- **Type status:** Paratype. **Occurrence:** recordedBy: SL Montgomery; individualCount: 1; sex: female; **Location:** islandGroup: Hawaiian Islands; island: Oahu; verbatimLocality: Mt. Tantalus; **Event:** verbatimEventDate: 1.i.1970; **Record Level:** institutionCode: BPBM
- **Type status:** Paratype. **Occurrence:** recordedBy: SL Montgomery; **Location:** islandGroup: Hawaiian Islands; island: Oahu; verbatimLocality: Palikea Gulch; **Event:** verbatimEventDate: 20.ii.1970; **Record Level:** institutionCode: UHEC
- **Type status:** Paratype. **Occurrence:** recordedBy: SL Montgomery; **Location:** islandGroup: Hawaiian Islands; island: Oahu; verbatimLocality: Waianae Kai Forest Reserve; verbatimElevation: 1800 ft; **Event:** verbatimEventDate: 31.v.1970; **Record Level:** institutionCode: UHEC
- **Type status:** Paratype. **Occurrence:** recordedBy: SL Montgomery; **Location:** islandGroup: Hawaiian Islands; island: Oahu; verbatimLocality: Puu Pane; **Event:** verbatimEventDate: 18.vi.1970; **Record Level:** institutionCode: UHEC
- **Type status:** Paratype. **Occurrence:** recordedBy: SL Montgomery; **Location:** islandGroup: Hawaiian Islands; island: Oahu; verbatimLocality: Opaeula; **Event:** verbatimEventDate: 20.vi-16.vii.1970; **Record Level:** institutionCode: UHEC
- **Type status:** Paratype. **Occurrence:** recordedBy: SL Montgomery; **Location:** islandGroup: Hawaiian Islands; island: Oahu; verbatimLocality: Puu Kaua; **Event:** verbatimEventDate: 4-27.viii.1970; **Record Level:** institutionCode: UHEC
- **Type status:** Paratype. **Occurrence:** recordedBy: SL Montgomery; **Location:** islandGroup: Hawaiian Islands; island: Oahu; verbatimLocality: East Makaleha; **Event:** verbatimEventDate: 7.xi.1970; **Record Level:** institutionCode: UHEC
- **Type status:** Paratype. **Occurrence:** recordedBy: SL Montgomery; **Location:** islandGroup: Hawaiian Islands; island: Oahu; verbatimLocality: Mokuleia; **Event:** verbatimEventDate: 23.xi.1970; **Record Level:** institutionCode: UHEC
- **Type status:** Paratype. **Occurrence:** recordedBy: SL Montgomery; **Location:** islandGroup: Hawaiian Islands; island: Oahu; verbatimLocality: Puu Kanehoa; **Event:** verbatimEventDate: 21.ii.1971; **Record Level:** institutionCode: UHEC
- **Type status:** Other material. **Occurrence:** recordedBy: DE Hardy; individualCount: 1; **Location:** islandGroup: Hawaiian Islands; island: Hawaii; verbatimLocality: Hualalai, N slope kipuka; verbatimElevation: 5000 ft; **Identification:** identifiedBy: K Arakaki; **Event:** eventDate: 7.x.1968; **Record Level:** institutionCode: BPBM
- **Type status:** Other material. **Occurrence:** recordedBy: SL Montgomery; **Location:** islandGroup: Hawaiian Islands; island: Maui; verbatimLocality: Kaupo Gap; verbatimElevation: 4800 ft; **Event:** verbatimEventDate: 21.iv.1971; **Record Level:** institutionCode: UHEC
- **Type status:** Paratype. **Occurrence:** recordedBy: SL Montgomery; **Location:** islandGroup: Hawaiian Islands; island: Oahu; verbatimLocality: Niu; verbatimElevation: 1600 ft; **Event:** verbatimEventDate: 11.vii.1971; **Record Level:** institutionCode: UHEC
- **Type status:** Other material. **Occurrence:** recordedBy: WM Giffard; **Location:** islandGroup: Hawaiian Islands; island: Hawaii; verbatimLocality: North Kona, Puuwaawaa; verbatimElevation: 3700 ft; **Event:** verbatimEventDate: no date given; **Record Level:** institutionCode: UHEC
- **Type status:** Other material. **Occurrence:** recordedBy: WC Gagne; individualCount: 1; **Location:** islandGroup: Hawaiian Islands; island: Oahu; verbatimLocality: Kipahulu Valley, nr. Puu Ahuula; verbatimElevation: 300 m; **Event:** eventDate: 23.vii.1980; **Record Level:** collectionID: 1980.322; institutionCode: BPBM
- **Type status:** Other material. **Occurrence:** recordedBy: WC Gagne; individualCount: 1; **Location:** islandGroup: Hawaiian Islands; island: Oahu; verbatimLocality: Kuliouou For. Res., Euphorbia celastroides; verbatimElevation: 609 m; **Event:** eventDate: 16.xi.1980; **Record Level:** collectionID: 1980.545; institutionCode: BPBM
- **Type status:** Other material. **Occurrence:** recordedBy: GM Nishida; individualCount: 1; **Location:** islandGroup: Hawaiian Islands; island: Oahu; verbatimLocality: Lualualei, Halona Valley, at MV light; verbatimElevation: 1420 ft; **Event:** eventDate: 19-20.i.1996; **Record Level:** institutionCode: BPBM
- **Type status:** Other material. **Occurrence:** recordedBy: KN Magnacca; individualCount: 1; **Location:** islandGroup: Hawaiian Islands; island: Oahu; verbatimLocality: Manoa Cliff Trail, in Freycinetia axil; verbatimElevation: 1800 ft; **Identification:** identifiedBy: PM O'Grady; **Event:** verbatimEventDate: 25.ii.2007; **Record Level:** institutionID: EMEC7356012; institutionCode: ECEM; collectionCode: 07-0176
- **Type status:** Other material. **Occurrence:** recordedBy: KN Magnacca; individualCount: 1; **Location:** islandGroup: Hawaiian Islands; island: Maui; verbatimLocality: Puu Kukui, Kahanaiki Gulch, sweeping Cyrtandra; verbatimElevation: 2100 ft; **Identification:** identifiedBy: PM O'Grady; **Event:** verbatimEventDate: 7.viii.2007; **Record Level:** institutionID: EMEC7356013; institutionCode: ECEM; collectionCode: 07-0819
- **Type status:** Other material. **Occurrence:** recordedBy: PM O'Grady, RT Lapoint, GM Bennett, NA Pantoja; individualCount: 1; **Location:** islandGroup: Hawaiian Islands; island: Hawaii; verbatimLocality: Hawaii Volcanoes National Park, Kipuka Puaulu; **Identification:** identifiedBy: PM O'Grady; **Event:** verbatimEventDate: 1.viii.2010; **Record Level:** institutionID: EMEC7356014; institutionCode: ECEM; collectionCode: 610.4

##### Ecological interactions

###### Feeds on

This species has been reared from rotting stems and bark of several native Hawaiian plant species, including *Pisonia*, *Charpentiera*, *Urera* and *Hibiscadelphus*.

###### Native status

endemic

##### Distribution

HAWAIIAN ISLANDS: Kauai, Oahu, Molokai, Maui, Hawaii

##### Notes

Hardy and Delfinado (1980) [original description; illustration of male genitalia (lateral, Oahu), male genitalia (lateral, Hawaii), egg]; Sabrosky (1989) [Australasian and Oceanian Catalog]; Nishida (2002) [Hawaiian Terrestrial Arthropod Checklist]; Evenhuis (2011) [http://hbs.bishopmuseum.org/aocat/asteiidae.html, Australasian and Oceanian Catalog, online version].

## Discussion

These revised numbers of single island endemics signifanctly change how our interpretations of diversification may be occurring, particularly in smaller, more recent radiations like *Asteia*. The prevailing dogma is that a population of one species colonizes a neighbor island, becomes reproductively isolated and, over time, diverges in allopatry, forming a distinct sister species. There are many examples of such divergence in the Hawaian *Drosophila* (*e.g.*, Bonacum et al. 2005). Taxa endemic to Maui Nui (the islands of Maui, Molokai, Lanai and Kahoolawae) often display a slightly different pattern due to the recent historical connections present between these islands in periods of low sea stands (Price and Elliott-Fisk 2004). If a species is present on more than one island, it is often the case that these islands are in the Maui Nui group. This is the case with two of the seven new island records we report in this paper. There is little evidence concerning the dispersal abilities of these taxa or the potential mechanism for inter-island transport. There are no flightless or brachypterous *Asteia* so they may be able to fly from island to island. They may also be blown from one island to another, as has been suggested for several other groups of Hawaiian Diptera (Nitta and O’Grady 2008, O'Grady et al. 2009, Zimmerman 1948). Additional work is clearly needed to better understand dispersal ability in various groups of Hawaiian arthropods.

Multiple lineages of broadly overlapping species suggest another process may be acting in groups such as *Dicranomyia* (Goodman and O'Grady 2013, Nitta and O’Grady 2008) and *Asteia*. In these groups divergence may take place on smaller geographic scales (*e.g.*, within an island) or be driven by adaptation to different ecological niches. Two species of Hawaiian *Asteia* have been reared from a number of native Hawaiian tree species (Table 2), suggesting an ecological role for divergence within this group. While some taxa (*e.g.*, *Asteia nudiseta* on *Suttonia* = *Myrsine*) have label data that indicate some association with a specific plant, we did not include them in Table 2 unless the author stated they were reared.

## Supplementary Material

XML Treatment for Asteia aberrans

XML Treatment for Asteia apicalis

XML Treatment for Asteia hawaiiensis

XML Treatment for Asteia mauiensis

XML Treatment for Asteia molokaiensis

XML Treatment for Asteia montgomeryi

XML Treatment for Asteia nudiseta

XML Treatment for Asteia palikuensis

XML Treatment for Asteia sabroskyi

## Figures and Tables

**Table 1.** **Distribution of *Asteia* species in Hawaii.** Status indicates whether the species are endemic (E), introduced (I) or adventive (A). Islands sampled are: Kauai (K), Oahu (O), Molokai (Mo), Lanai (L), Maui (Ma), and Hawaii (H). Presence of a species on a given island is designated by an X. An asterisk denotes a new island record for the species.

Species	Status	NWHI	K	O	Mo	L	Ma	H
*Asteia aberrans*	E	X						
*Asteia apicalis*	E						X	X
*Asteia hawaiiensis*	E						X*	X
*Asteia mauiensis*	E			X*			X	
*Asteia molokaiensis*	E				X	X*	X*	
*Asteia montgomeryi*	E							X
*Asteia nudiseta*	E			X				
*Asteia palikuensis*	E					X*	X	X*
*Asteia sabroskyi*	E		X	X	X		X	X*

**Table 2.** **Rearing records for endemic Hawaiian *Asteia*.** Tabular summary of rearing records for Hawaiian *Asteia* (Hardy and Delfinado 1980).

Species	Island	Record
*Asteia montgomeryi*	Hawaii	*Erythrina sandwichensis*, rotting stems
*Asteia sabroskyi*	Oahu	*Pisonia* sp., rotting stem and bark
	Oahu	*Urera* sp., rotting stem
	Oahu	*Charpentiera* sp., rotting stem
	Oahu	*Hibiscadelphus* sp., rotting bark
	Maui	*Pisonia* sp., rotting stem
